# An OSMAC Strategy for the Production of Antimicrobial Compounds by the Amazonian Fungi *Talaromyces pinophilus* CCM-UEA-F0414 and *Penicillium paxilli* CCM-UEA-F0591

**DOI:** 10.3390/antibiotics14080756

**Published:** 2025-07-27

**Authors:** Cleudiane Pereira de Andrade, Caroline Dutra Lacerda, Raíssa Assímen Valente, Liss Stone de Holanda Rocha, Anne Terezinha Fernandes de Souza, Dorothy Ívila de Melo Pereira, Larissa Kirsch Barbosa, Cleiton Fantin, Sergio Duvoisin Junior, Patrícia Melchionna Albuquerque

**Affiliations:** 1 Grupo de Pesquisa Química Aplicada à Tecnologia, Escola Superior de Tecnologia, Universidade do Estado do Amazonas, Manaus 69055-035, Brazil; cleudiane.andrade@hotmail.com (C.P.d.A.); cdutralacerda@gmail.com (C.D.L.); contatoraissavalente@gmail.com (R.A.V.); lissandroshr@gmail.com (L.S.d.H.R.); anne.fernandes13@gmail.com (A.T.F.d.S.); dorothyivila@gmail.com (D.Í.d.M.P.); sjunior@uea.edu.br (S.D.J.); 2 Programa de Pós-Graduação em Biodiversidade e Biotecnologia da Rede Bionorte, Universidade do Estado do Amazonas, Escola Superior de Tecnologia, Manaus 69055-035, Brazil; 3 Programa Multicêntrico de Pós-Graduação em Bioquímica e Biologia Molecular, Universidade do Estado do Amazonas, Manaus 69065-001, Brazil; cfantin@uea.edu.br; 4 Programa de Pós-Graduação em Biotecnologia e Recursos Naturais da Amazônia, Universidade do Estado do Amazonas, Manaus 69050-010, Brazil; lkirsch@uea.edu.br

**Keywords:** antimicrobial activity, phenolic compounds, Amazon, secondary metabolites, endophytic fungi

## Abstract

**Background/Objectives:** The emergence of antimicrobial resistance represents a critical global health threat, requiring the discovery of novel bioactive compounds. Fungi from Amazonian biodiversity are promising sources of secondary metabolites with potential antimicrobial activity. This study aimed to investigate the production of antimicrobial compounds by two Amazonian fungal strains using the OSMAC (One Strain–Many Compounds) approach. **Methods:** Two fungal strains, *Talaromyces pinophilus* CCM-UEA-F0414 and *Penicillium paxilli* CCM-UEA-F0591, were cultivated under five distinct culture media to modulate secondary metabolite production. Ethyl acetate extracts were prepared and evaluated for antimicrobial activity against Gram-positive and Gram-negative bacteria, as well as pathogenic yeasts. Chemical characterization was performed using thin-layer chromatography (TLC), Fourier Transform Infrared Spectroscopy (FTIR), Ultraviolet–Visible (UV-Vis) spectroscopy, and Ultra-High-Performance Liquid Chromatography with Diode Array Detection (uHPLC-DAD). **Results:** The extracts exhibited significant antimicrobial activity, with minimum inhibitory concentrations (MICs) ranging from 78 to 5000 µg/mL. Chemical analyses revealed the presence of phenolic compounds, particularly caffeic and chlorogenic acids. Variations in the culture media substantially affected both the metabolite profiles and antimicrobial efficacy of the extracts. **Conclusions:** The OSMAC strategy effectively enhanced the metabolic diversity of the Amazonian fungal strains, leading to the production of bioactive metabolites with antimicrobial potential. These findings support the importance of optimizing culture conditions to unlock the biosynthetic capacity of Amazonian fungi as promising sources of antimicrobial agents.

## 1. Introduction

Since the introduction of the first antibiotics in the mid-20th century, numerous lives have been saved. However, the emergence of resistant pathogens continues to pose significant health challenges. Current antibiotic development has not kept pace with the rapid evolution of antimicrobial resistance, creating a pressing need for new therapeutic compounds [1,2,3].

Fungal natural products represent a promising yet underexplored reservoir of bioactive secondary metabolites, particularly in biodiverse ecosystems such as the Amazon rainforest [4,5]. This region hosts an immense diversity of fungal species, many of which remain undescribed or poorly characterized. Among these, the genera *Talaromyces* and *Penicillium* stand out due to their widespread distribution and remarkable ability to biosynthesize structurally diverse secondary metabolites. Both genera have been recognized for their capacity to produce antimicrobial, anticancer, and immunosuppressive compounds, highlighting their relevance in biotechnological research. While *Penicillium* is historically known for the discovery of penicillin, *Talaromyces* has more recently gained attention for its novel bioactive metabolites. Despite their potential, the Amazonian representatives of these genera remain largely untapped as sources of antimicrobial agents [5].

These bioactive metabolites serve ecological functions related to defense and adaptation and include diverse classes such as alkaloids, terpenoids, and phenolic compounds [6]. Optimizing their production is crucial for advancing their biotechnological application [7,8]. Importantly, fungal secondary metabolite production is often tightly regulated by environmental and nutritional factors. Under standard laboratory conditions, many biosynthetic gene clusters (BGCs) remain transcriptionally silent, limiting the expression of potentially valuable metabolites [9,10]. Therefore, strategies to stimulate the activation of silent BGCs are essential for uncovering the full metabolic potential of these organisms. In this context, the One Strain–Many Compounds (OSMAC) approach provides a simple yet powerful framework for diversifying secondary metabolite production by varying culture conditions [11]. This technique can be applied through co-cultivation, variation in cultivation conditions, and/or epigenetic modification, exposing the fungi’s critical natural biosynthetic capacity [12,13,14].

The One Strain–Many Compounds (OSMAC) approach, pioneered by Bode et al. [15], systematically varies cultivation parameters, such as nutrient composition, aeration, vessel type, and the addition of metabolic modulators, to stimulate silent biosynthetic pathways. This methodology has demonstrated significant success in broadening microbial chemical diversity and facilitating novel bioactive compound discovery [15,16,17]. It is a valuable tool for overcoming the limitations of laboratory cultivation experiments and represents a powerful and effective strategy for discovering novel metabolites with biological potential, especially considering that secondary metabolites from these Amazonian fungi exhibit numerous biological activities due to their structural diversity and have emerged as an important source of antimicrobial compounds.

Species of the genus *Penicillium* are well known for their ability to produce a wide array of antimicrobial metabolites, making them a valuable resource in the search for novel therapeutic agents. A strain of *Penicillium raistrichii* was subjected to the OSMAC strategy by altering the fermentation conditions from shaking to a static liquid environment, promoting the accumulation of four novel secondary metabolites, including three β-carbolines and one 2-quinolinone, as well as a new natural compound, 2-quinolinone, and five previously known alkaloids. All identified compounds exhibited clear antimicrobial activity against at least one of the tested pathogens, with compound 1 showing notable MIC values of 8, 5, and 2 μg/mL against *Staphylococcus aureus*, *Escherichia coli*, and *Candida albicans*, respectively [17].

Despite the significant biodiversity of fungi in the Amazon biome and their potential as sources of novel bioactive compounds, relatively few studies have explored Amazonian fungal isolates in the context of secondary metabolite production under varied cultivation conditions. This represents a substantial gap in natural product research and drug discovery. In this context, the present study aims to address this gap by applying the OSMAC approach to two filamentous fungi—*Talaromyces pinophilus* CCM-UEA-F0414 and *Penicillium paxilli* CCM-UEA-F0591—isolated from native Amazonian plants. The objective is to evaluate how variations in culture media influence the biosynthesis of antimicrobial secondary metabolites in these strains. This investigation not only contributes to the discovery of new bioactive compounds from Amazonian fungi but also enhances our understanding of how culture conditions modulate fungal metabolic expression, offering valuable insights for future bioprospecting and biotechnological applications.

## 2. Results

### 2.1. Identification of Fungal Strains

**Figure 1:**
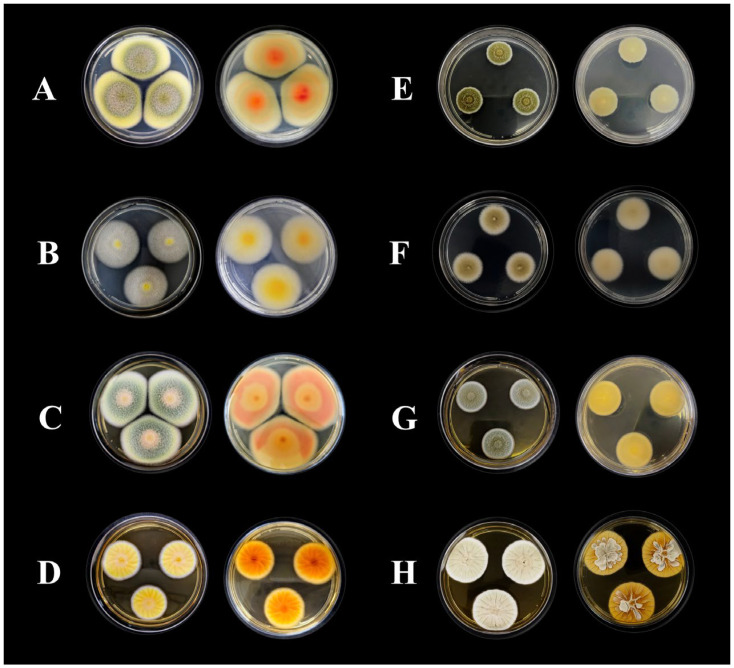
Macroscopic characteristics (obverse and reverse) of fungal colonies after 7 days of incubation at 28 °C in absence of light: CCM-UEA-F0414 grown on PDA (**A**), CYA (**B**), MEA (**C**), and YES (**D**) and CCM-UEA-F0591 grown on PDA (**E**), CYA (**F**), MEA (**G**), and YES (**H**).

Following an initial screening of 28 Amazonian fungal species, 2 strains were selected for this study based on their pronounced antimicrobial compound production. They were cultivated on four different solid media to assess their macromorphological characteristics, which revealed distinct features (Figure 1).

The morphological characterization of strain CCM-UEA-F0414 revealed colonies on PDA medium (Figure 1A) reaching 49 mm in diameter after 7 days of incubation, exhibiting dark olive green to forest green margins and yellowish-green centers, with tomato and silk-corn hues on the reverse. Colonies presented a cottony texture, regular margins, flat elevation, and no exudate or pigment production. On CYA medium (Figure 1B), colonies reached 37 mm in diameter with lemon yellow surfaces and golden to khaki reverses, showing a cottony texture, regular margins, flat elevation, and no pigment or exudate. No growth was observed at 5 or 37 °C. On MEA medium (Figure 1C), colonies reached 49 mm in diameter with light salmon, military green, yellowish-green, and white hues from the margin to center, and brown chocolate tones on the reverse; the texture remained cottony with flat elevation and no pigment or exudate. On YES medium (Figure 1D), colonies reached 28 mm, with yellow and white surfaces and dark orange to yellow reverses. Colonies showed a velvety texture, regular margins, wrinkled elevation, transparent exudate droplets, and no extracellular pigment.

For strain CCM-UEA-F0591, colonies on PDA (Figure 1E) reached 20 mm, displaying military green and white surfaces with white reverses, powdery to sandy texture, regular margins, flat elevation, and no exudate or pigment production. On CYA medium (Figure 1F), colonies reached 28 mm in diameter, showing dark khaki and white surfaces with Navajo white reverses, cottony texture, regular margins, flat elevation, and no exudate or pigment production. No growth was observed at 5 or 37 °C. On MEA medium (Figure 1G), colonies reached 25 mm with military green and white surfaces, white reverses, and a powdery/sandy texture, with flat elevation and no exudate or pigment. On YES medium (Figure 1H), colonies reached 38 mm, displaying grayish-green and white surfaces and reverses, velvety texture, regular margins, crateriform wrinkled elevation, and no exudate or pigment production.

**Figure 2:**
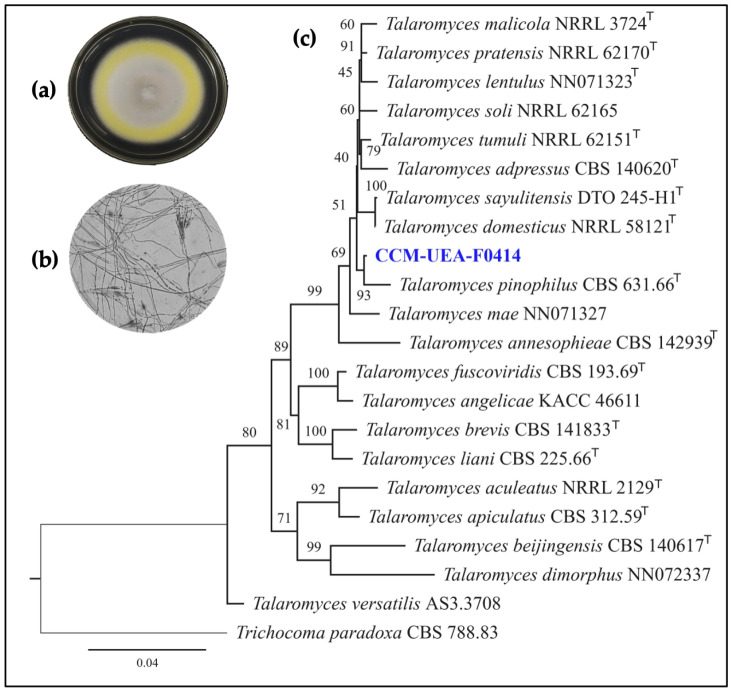
(**a**) Macroscopic morphology of CCM-UEA-F0414 colonies on PDA medium after 7 days of incubation at 28 °C. (**b**) Microscopic features on MEA medium observed at 1000× magnification after 7 days. (**c**) Maximum likelihood (ML) phylogenetic tree based on concatenated ITS and *rpb2* sequences, showing taxonomic position of endophytic fungus CCM-UEA-F0414 (highlighted in blue), isolated from stem cortex of *Myrcia guianensis* (Myrtaceae). Bootstrap support values are indicated at each node, and scale bar represents number of substitutions per site. Tree was rooted with *Trichocoma paradoxa* CBS 788.83, and type strains are marked with superscript “T”.

The molecular identification of the endophytic fungus CCM-UEA-F0414 was performed by amplifying the internal transcribed spacer (ITS) region and the partial second largest subunit of DNA-directed RNA polymerase II (*rpb2*), yielding 93% bootstrap support with the neotype strain of *Talaromyces pinophilus* CBS 631.66. Twenty representative isolates were used for constructing the phylogenetic tree of the genus *Talaromyces* using combined ITS and *rpb2* locus analysis (Figure 2). The best-fit model according to the Bayesian Information Criterion (BIC) was TNe + G4 for both partitions.

**Figure 3:**
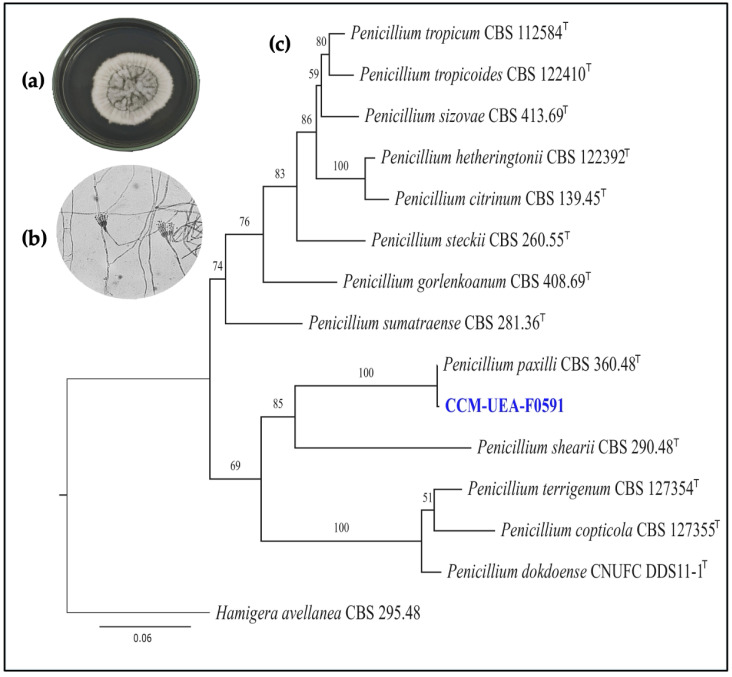
(**a**) Macroscopic morphology of CCM-UEA-F0591 colonies on PDA medium after 7 days of incubation at 28 °C. (**b**) Microscopic features on MEA medium observed at 1000× magnification after 7 days. (**c**) Maximum likelihood (ML) phylogenetic tree based on concatenated ITS, *rpb2*, and *tub2* sequences, showing taxonomic position of endophytic fungus CCM-UEA-F0591 (highlighted in blue), isolated from branch of *Aniba canelilla* (Lauraceae). Tree was rooted with *Hamigera avellanea* CBS 295.48. Scale bar represents 0.06 substitutions per site, bootstrap support values are indicated, and type strains are marked with superscript “T”.

The epiphytic fungus CCM-UEA-F0591 was identified by amplifying the ITS, the partial beta-tubulin gene (*tub2*), and *rpb2*, yielding 100% bootstrap support with the neotype strain of *Penicillium paxilli* CBS 360.48. Thirteen representative isolates (*Copticolarum*, *Sumatraensia*, *Sheariorum*, *Paxillorum*, and *Citrina* series) were used for constructing the *Penicillium* phylogenetic tree using combined ITS, *rpb2*, and *tub2* loci (Figure 3). The best-fit models according to the BIC were TIM2 + F + I (partition 1), TNe + G4 (partition 2), and TNe + G4 (partition 3). GenBank accession numbers for the fungal isolates used in this study are provided in Table 1.

### 2.2. Antimicrobial Activity

The ethyl acetate extracts obtained from *T. pinophilus* CCM-UEA-F0414 and *P. paxilli* CCM-UEA-F0591 cultivated on different media were evaluated for antimicrobial activity (Table 2). Extracts from both fungi inhibited the growth of Gram-positive and Gram-negative bacteria as well as pathogenic yeasts, exhibiting varying susceptibility responses. Furthermore, variations in the cultivation media led to differences in antimicrobial activity intensity, with minimum inhibitory concentrations ranging from 78 to 5000 µg/mL for both fungal extracts. These values are significant for crude fungal extracts, considering that no purified compounds were tested.

### 2.3. Chemical Characterization of Fungal Extracts

**Figure 4:**
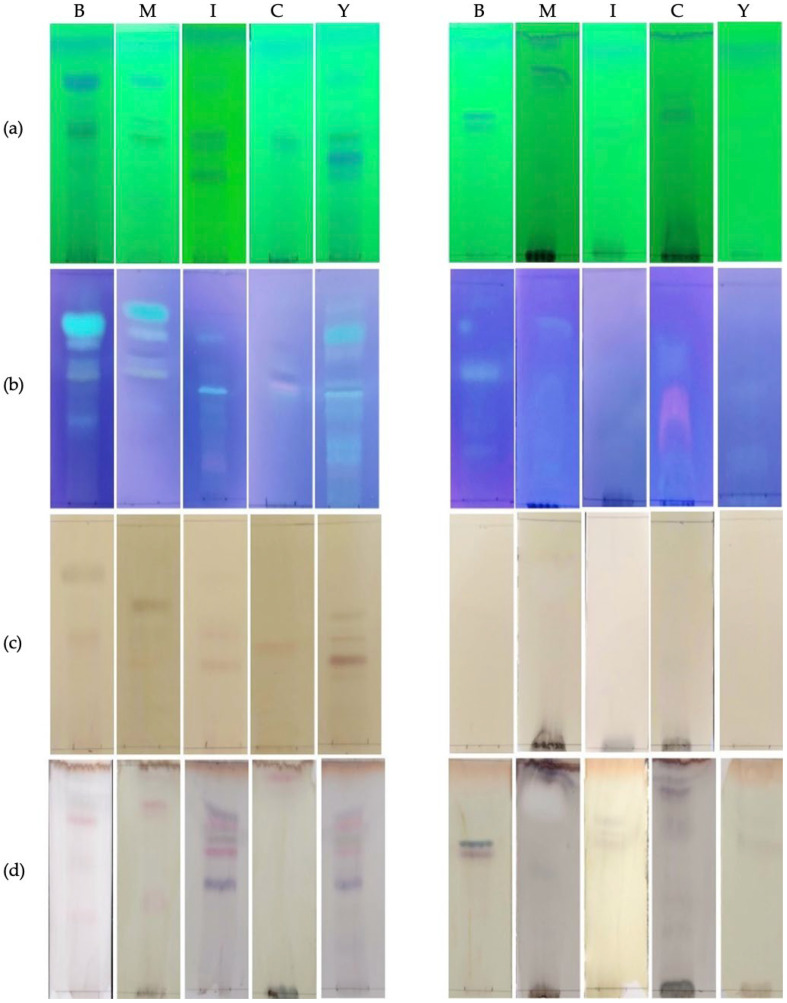
TLC of fungal extracts produced by *T. pinophilus* CCM-UEA-F0414 (1) and *P. paxilli* CCM-UEA-F0591 (2), obtained after cultivation on BDL (B), modified ISP2 (I), YES (Y), modified Czapek (C), or malt (M) medium. (**a**) UV light at 254 nm, indicating presence of conjugated double bonds; (**b**) stained with aluminum chloride and exposed under UV light at 365 nm, indicating presence of flavonoids; (**c**) stained with ferric chloride, indicating presence of phenolic compounds; and (**d**) stained with vanillin/H_2_SO_4_, indicating presence of terpenes (purple spots) and flavonoids (red spots).

Each extract was analyzed by thin-layer chromatography (TLC) to identify the major classes of metabolites present (Figure 4). In the plates developed with aluminum chloride and observed under UV light at 365 nm, fluorescent yellow bands indicated the presence of flavonoids, while blue bands suggested the presence of phenolic acids [18]. The TLC plates stained with aluminum chloride, ferric chloride, and vanillin revealed that the extracts of *T. pinophilus* CCM-UEA-F0414 and *P. paxilli* CCM-UEA-F0591 are rich in phenolic compounds. No bands were observed on the TLC plates treated with Dragendorff reagent or cerium sulfate, indicating the absence of alkaloids and terpenes, respectively.

**Figure 5:**
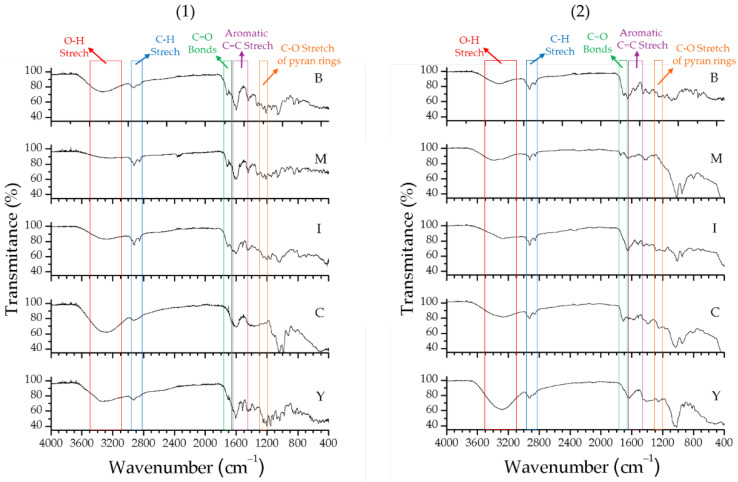
FTIR spectra of fungal extracts produced by *T. pinophilus* CCM-UEA-F0414 (**1**) and *P. paxilli* CCM-UEA-F0591 (**2**) after cultivation in BDL (B), modified ISP2 (I), YES (Y), modified Czapek (C), and malt (M) media.

The infrared spectra of the extracts from both fungi corroborate the TLC results, indicating a profile suggestive of phenolic compounds. Absorption in the range of 3500–3100 cm^−1^ corresponds to O-H stretching from different chemical environments; peaks near 2900 cm^−1^ correspond to C-H bonds; aromatic C=C stretching appears between 1611 and 1444 cm^−1^; and absorption around 1284–1283 cm^−1^ reflects the C-O stretching of pyran rings, typical of flavonoid C-rings. Absorption bands at 1670–1720 cm^−1^ indicate C=O bonds associated with carboxylic acids [19]. The main absorption bands observed in the FTIR spectra (Figure 5) confirm the abundant presence of phenolic compounds, including flavonoids and phenolic acids, in the evaluated extracts.

**Figure 6:**
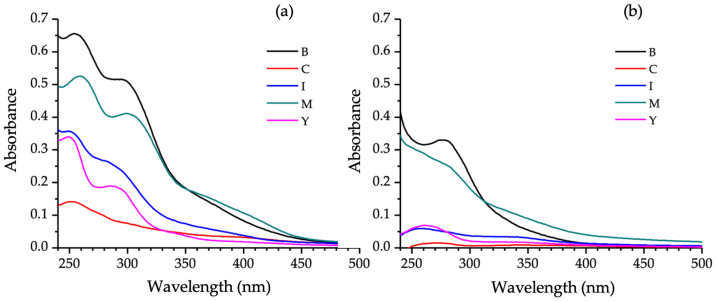
UV-Vis absorption spectra of fungal extracts produced by *T. pinophilus* CCM-UEA-F0414 (**a**) and *P. paxilli* CCM-UEA-F0591 (**b**) after cultivation on BDL (B), modified ISP2 (I), YES (Y), modified Czapek (C), or malt (M) medium.

The UV–visible absorption spectra of each extract are shown in Figure 6. The high absorption in the UV region supports the presence of phenolic compounds, as multiple conjugated bonds in their structures make them strong chromophores with characteristic absorption in this region [20]. The presence of flavonoids is suggested by the two main absorption bands: Band I (320–385 nm), corresponding to ring B absorption, and Band II (250–285 nm), corresponding to ring A absorption. None of the evaluated extracts showed characteristic anthocyanin bands (450–560 nm) [21], corroborating the TLC results revealed with aluminum chloride.

**Figure 7:**
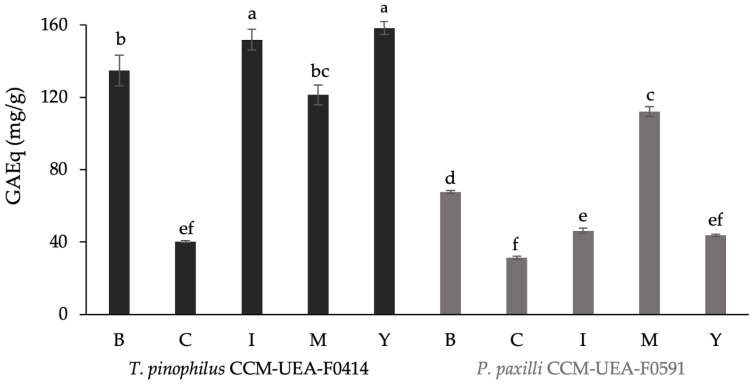
Total phenolic content in fungal extracts produced by *T. pinophilus* CCM-UEA-F0414 and *P. paxilli* CCM-UEA-F0591 following cultivation in BDL (B), modified ISP2 (I), YES (Y), modified Czapek (C), or malt (M) medium, expressed in mg of gallic acid equivalents per gram of dry extract (mg GAEq/g). Values that share same letters have no significant differences between them according to Tukey test (*p* < 0.05).

Figure 7 presents the total phenolic content detected in each extract. Regardless of the culture medium used, the extracts from *T. pinophilus* CCM-UEA-F0414 contained higher amounts of phenolic compounds compared to *P. paxilli* CCM-UEA-F0591. The medium yielding the highest phenolic content was YES for F0414 and malt for F0591, while Czapek resulted in the lowest production for both fungi.

**Figure 8:**
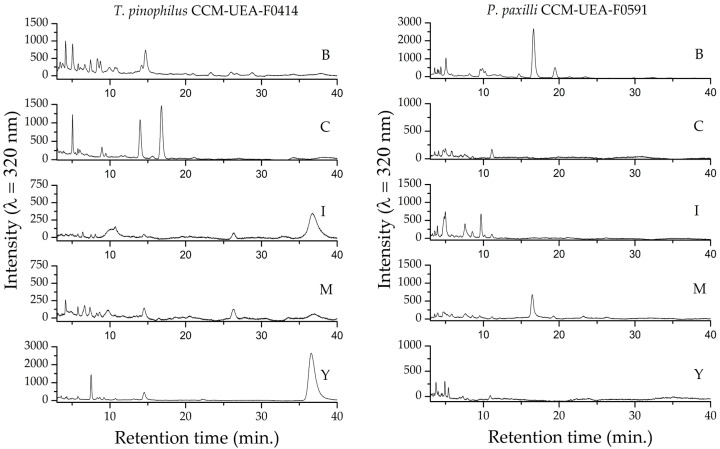
Chromatographic profiles (uHPLC-DAD at 320 nm) of fungal extracts produced by *T. pinophilus* CCM-UEA-F0414 and *P. paxilli* CCM-UEA-F0591 following cultivation in BDL (B), modified ISP2 (I), YES (Y), modified Czapek (C), or malt (M) medium, illustrating compound diversity.

The chromatographic profiles of the fungal extracts, determined by uHPLC-DAD (Figure 8), are consistent with the results obtained by TLC, FTIR, and UV-Vis, indicating the presence of phenolic compounds. The chromatograms were compared to standards of caffeic acid (retention time: 5.05 min) and chlorogenic acid (retention time: 3.67 min). These results are summarized in Table 3, and the corresponding chromatograms are provided in Supplementary Figures S1 and S2. Several additional peaks were also observed across all extracts, suggesting the presence of various other compounds besides the evaluated standards. Differences in the chromatographic profiles demonstrate that the culture medium influences the metabolic profile of the evaluated fungi.

## 3. Discussion

Fungi are known for their remarkable ability to biosynthesize a broad spectrum of secondary metabolites with diverse chemical structures and bioactivities, including antibacterial, antifungal, anticancer, and immunosuppressive effects. The expression of these metabolites is often tightly regulated by environmental factors, including the composition of the growth medium, temperature, and pH, which makes their discovery and production highly variable and challenging [22]. Although classical fermentation and extraction techniques have yielded numerous therapeutic agents, these methods tend to repeatedly isolate well-characterized compounds. Consequently, new strategies are required to uncover previously unexplored metabolic capacities.

In this study, the OSMAC (One Strain–Many Compounds) approach was applied to two Amazonian fungi, *Talaromyces pinophilus* CCM-UEA-F0414 and *Penicillium paxilli* CCM-UEA-F0591, isolated from the native plant species *Myrcia guianensis* and *Aniba canelilla*, respectively [23,24]. These plant species are known for their medicinal properties and ecological relevance in the Amazon biome. Despite their significance, few studies have explored the endophytic or epiphytic fungi associated with these plants, particularly regarding their capacity to produce bioactive metabolites [25].

The use of five distinct culture media allowed us to examine the impact of nutrient composition on secondary metabolite production and bioactivity. The OSMAC strategy revealed distinct metabolic profiles that varied according to the growth medium. While simple in design, this technique represents a powerful method for maximizing chemical diversity and guiding subsequent compound isolation efforts [15].

Among the tested pathogens, *C. albicans* exhibited the highest sensitivity to fungal extracts. Specifically, *T. pinophilus* grown on YES (Y) and *P. paxilli* on BDL (B) produced extracts with MIC values of 78 µg/mL, comparable to that of the positive control terbinafine (50 µg/mL). For *P. paxilli* cultivated on modified ISP2 (I), the MIC against *S. epidermidis* was 313 µg/mL, similar to that of the control levofloxacin (250 µg/mL). Against *E. faecalis*, extracts from the malt (M) medium showed MICs of 2500 µg/mL, which was comparable to 1953 µg/mL for the standard. These data highlight the relevance of crude fungal extracts as potential antimicrobial sources.

It is well established that the antimicrobial properties of phenolic acids are attributed to their ability to disrupt microbial membranes, chelate metal ions, and interfere with critical enzymes involved in cell wall synthesis [26,27]. The selective inhibition of *C. albicans* and *S. pyogenes* by the YES and BDL extracts is consistent with these mechanisms, as these microorganisms are particularly susceptible to oxidative stress and membrane-targeting agents. In contrast, the reduced activity of the extracts against Gram-negative bacteria such as *K. pneumoniae* and *E. coli* may be attributed to the intrinsic resistance conferred by their outer membrane barrier, which restricts the penetration of hydrophilic phenolic compounds. Interestingly, *P. aeruginosa*, which also possesses this barrier, was inhibited at moderate concentrations, suggesting the presence of additional bioactive metabolites beyond phenolic acids. Among the tested pathogens, *K. pneumoniae* exhibited the highest resistance, indicating species-specific susceptibility patterns.

The antimicrobial profile varied significantly with the culture medium, confirming the influence of nutrient composition on secondary metabolism. For *T. pinophilus,* modified Czapek (C) medium enhanced activity against a broad pathogen panel, while YES (Y) demonstrated strong antifungal action. In the case of *P. paxilli*, malt (M) medium supported activity against 11 pathogens, but the BDL (B) extract was the most effective against C. albicans. These findings underscore the importance of culture conditions in modulating metabolite biosynthesis. Among the tested extracts, those derived from cultures grown in YES and BDL media exhibited the highest antimicrobial efficacy, particularly against *C. albicans.* These media are notably rich in sugars and yeast extract, respectively—both of which are recognized as metabolic modulators in fungi, capable of stimulating the shikimate pathway and related biosynthetic routes [28,29,30]. The detection of caffeic acid and chlorogenic acid—compounds widely known for their antimicrobial properties—corroborates the observed biological activity [28,31].

Chemical analysis using TLC, FTIR, UV–Vis, and uHPLC-DAD confirmed the presence of phenolic compounds, a class of secondary metabolites known for their antimicrobial, antioxidant, and cytotoxic properties [32,33,34]. Notably, phenolic acids were previously identified in *Penicillium citrinum* extracts, including one with selective antimicrobial activity against *C. albicans* (8 µg/mL), *S. aureus* (128 µg/mL), and other pathogens [35]. Furthermore, these analyses confirmed the enrichment of hydroxylated aromatic compounds in the most active extracts, consistent with the phenolic nature of caffeic and chlorogenic acids. Notably, the TLC profiles revealed additional similar bands across media, suggesting the co-occurrence of structurally related analogs—such as ferulic and protocatechuic acids—which have been previously reported in *Penicillium* and *Talaromyces* species under specific culture conditions.

Variation in phenolic content and metabolite composition was closely tied to the culture medium, with differences in carbon and nitrogen sources likely playing a central role. It is well established that carbon sources provide not only energy but also structural precursors for secondary metabolite biosynthesis, while nitrogen sources regulate key metabolic pathways [36,37,38].

The production of caffeic acid, chlorogenic acid, and related analogs by species of *Penicillium* and *Talaromyces* is not as extensively documented as in plants. However, several studies have reported the occurrence of phenolic compounds (such as ferulic acid, caffeic acid, and protocatechuic acid) in these genera, particularly under stress conditions or when cultivated in media that induce secondary metabolism [39,40,41].

These compounds belong to the hydroxycinnamic acid class and are traditionally described as derivatives of the shikimate pathway, which is responsible for the biosynthesis of aromatic amino acids such as phenylalanine and tyrosine [42]. In filamentous fungi, caffeic acid can be synthesized from phenylalanine via the enzymatic action of phenylalanine ammonia-lyase (PAL) and cinnamic acid 4-hydroxylase [41], whereas chlorogenic acid results from the esterification of caffeic acid with quinic acid [43]. Although this pathway is better characterized in plants, genomic studies have shown that filamentous fungi harbor homologous genes related to the shikimate pathway [41,44]. Alternative biosynthetic routes, such as polyketide modifications, have also been proposed, though they are less common for these specific compounds [45].

The presence of genes related to the shikimate pathway in the genomes of Talaromyces and Penicillium species supports their biosynthetic capacity to produce phenolic bioactive compounds, contributing to the chemical diversity observed in the extracts. This diversity likely reflects the fungi’s inherent metabolic potential, which is significantly influenced by cultivation conditions.

While the detection of caffeic and chlorogenic acids provides a partial explanation for the antimicrobial activity observed, the diversity of chromatographic peaks and variations across culture media suggest the presence of additional compounds, possibly acting in synergy. Indeed, previous studies have demonstrated that mixtures of phenolics can exhibit enhanced antimicrobial activity through additive or synergistic mechanisms [39,42]. Overall, this study reinforces the potential of Amazonian fungi as sources of novel antimicrobials. The combination of the OSMAC approach and comprehensive bioassays provided insights into how culture conditions affect the biosynthesis and bioactivity of fungal metabolites. Our findings highlight *T. pinophilus* CCM-UEA-F0414 and *P. paxilli* CCM-UEA-F0591 as promising candidates for future bioprospecting efforts and suggest that media optimization could lead to the discovery of new compounds active against resistant microbial pathogens.

## 4. Materials and Methods

### 4.1. Fungi

The fungal strains used in this study are deposited in the Microbiological Collections Center of the State University of Amazonas (CCM/UEA) and stored according to the Castellani method [46]. Strain CCM-UEA-F0414 was isolated as an endophyte from the stem cortex of *Myrcia guianensis* collected in São Pedro Community, Santarém, Pará, Brazil (02°32′08.9″ S, 54°54′23.9″ W). Strain CCM-UEA-F0591 was isolated as an epiphyte from the leaf surface of *Aniba canelilla* in Adolpho Ducke Forest Reserve, Manaus, Amazonas, Brazil (02.93071° S, 59.97544° W). Access to genetic heritage was registered in the National System for the Management of Genetic Heritage and Associated Traditional Knowledge under codes A2CE6A6 and A6869EA.

The strains were reactivated by inoculating a fragment from stock cultures onto potato dextrose agar (Kasvi, Pinhais, Brazil) and incubated in a microbiological incubator (Novatécnica, Piracicaba, Brazil) at 28 ± 2 °C for 7 days in the absence of light.

### 4.2. Fungal Identification

#### 4.2.1. Morphological Characterization

The isolates were inoculated at three points on the following media: Czapek Yeast Autolysate Agar (CYA), Yeast Extract Sucrose Agar (YES), Malt Extract Agar (MEA), and potato dextrose agar (PDA) (Kasvi, Pinhais, Brazil) [47]. Plates were incubated for 7 days at 25 °C and for 7 days at 30 °C (CYA plates only). After incubation, macromorphological characteristics were evaluated, including colony diameter, texture, topography, margin features, conidia coloration, surface and reverse colors, and soluble pigment production.

Microscope slides were prepared from MEA cultures. The slides were stained with lactophenol blue (Sigma-Aldrich, Darmstadt, Germany) and examined under a light microscope (Nikon, Tokyo, Japan). Micromorphological features were recorded, including conidiophore branching patterns; stipe dimension, shape, and texture; and ornamentation of stipes and conidia [48].

#### 4.2.2. Molecular Identification

Molecular identification was performed by sequencing the internal transcribed spacer (ITS) regions, β-tubulin (*tub2*), and partial DNA-directed RNA polymerase II subunit (*rpb2*). Genomic DNA was extracted using a modified CTAB 2% protocol [49].

PCR amplification (15 µL final volume) included the following: 3 mM MgCl_2_, 0.2 mM dNTPs, 1X reaction buffer, 0.2 µM of each forward and reverse primer, 1 U Taq polymerase, and 50 ng of fungal genomic DNA [50]. The annealing temperatures and sequences of primers (*Its*1/*Its*4, Bt-2a/Bt-2b, and RPB2-6F/fRPB2-7cR) used in this study are listed in Table 4.

The amplification protocol consisted of the following steps: an initial denaturation cycle at 95 °C for 5 min, followed by 35 cycles of denaturation at 95 °C for 30 s, annealing at 58 °C or 62 °C (depending on the primer pair) for 30 s, and extension at 72 °C for 1 min, followed by a final extension at 72 °C for 5 min.

PCR products were purified using the PureLink™ kit (Thermo Fisher Scientific, Waltham, MA, USA), and sequencing was performed on an ABI 3500xl Genetic Analyzer (Applied Biosystems, Foster City, CA, USA). Sequences were checked, aligned, edited, and analyzed using Bioedit v.7.2.6 software [54]. Preliminary identification was performed by comparison to reference sequences deposited in GenBank using BLASTN (NCBI, www.ncbi.nlm.nih.gov). Sequences showing high similarity with type and representative strains were selected for phylogenetic analysis and deposited in GenBank.

#### 4.2.3. Phylogenetic Analysis

The quality of obtained sequences was assessed using Phred (http://lbi.cenargen.embrapa.br/phph/ (accessed on 13 March 2025)). Sequences were checked, aligned, and analyzed with Bioedit v.7.2.6 [54]. Reference sequences from GenBank were used for the preliminary identification and selection of type specimens for phylogenetic inference. The sequences generated in this study were deposited in GenBank (Supplementary Materials).

For phylogenetic tree construction, sequences were aligned using MAFFT v.7 (https://mafft.cbrc.jp/alignment/software/ (accessed on 16 April 2025)) [55]. Locus concatenation was performed using MEGA (version X) [56]. Phylogenetic reconstruction was conducted using maximum likelihood (ML) in IQ-TREE (http://iqtree.cibiv.univie.ac.at/ (accessed on 16 April 2025)) [57]. ML analysis included 1000 bootstrap replicates using all sites, with evolutionary models previously estimated by ModelFinder [58]. Trees were visualized with FigTree v.1.4.4 (http://tree.bio.ed.ac.uk/software/figtree/ (accessed on 16 April 2025)) and edited using Inkscape (version 1.4.2).

### 4.3. Production and Extraction of Fungal Metabolites

To evaluate metabolite production via the OSMAC approach, a conidial suspension (10^6^ conidia/mL) was prepared under aseptic conditions. Aliquots of 300 µL were inoculated into Erlenmeyer flasks containing 150 mL of different culture media (Table 5), adjusted to pH 6.0, and incubated statically at 28 ± 2 °C for 14 days. After incubation, metabolites were extracted by adding an equal volume of ethyl acetate (P.A.; Química Credie, Manaus, Brazil) for 4 h (28 °C, 120 rpm). The mixture was filtered under vacuum to remove the mycelium, and the organic phase was collected using a Buchner funnel. The solvent was evaporated under reduced pressure in a rotary evaporator, and the crude extracts were stored at 4 °C for further analyses [4].

### 4.4. Antimicrobial Activity Assay

The antimicrobial activity of the extracts was evaluated by the microdilution method following Clinical and Laboratory Standards Institute (CLSI) guidelines [59]. TTC (2,3,5-triphenyltetrazolium chloride) was used for antifungal assays and resazurin for antibacterial assays. Pathogenic microorganisms were obtained commercially from Cefar Diagnóstica Ltda. (São Paulo, SP, Brazil): *Aspergillus brasiliensis* CCCD-AA001, *Candida albicans* CCCD-CC001, *C. tropicalis* CCCD-CC002, *C. parapsilosis* CCCD-CC004, *Escherichia coli* CCCD-E005, *Staphylococcus aureus* CCCD-S009, *S. epidermidis* CCCD-S010, *S. pyogenes* CCCD-S012, *Pseudomonas aeruginosa* CCCD-P004, *Enterococcus faecalis* CCCD-E002, *Salmonella enterica* CCCD-S003, *Bacillus subtilis* CCCD-B005, *Klebsiella pneumoniae* CCCD-K003, and *Streptococcus mutans* NCTC10449. Bacteria were maintained on Mueller–Hinton agar and fungi on Sabouraud agar (Kasvi, Pinhais, Brazil).

In 96-well microplates, 100 µL of fungal extract at different concentrations (10,000; 5000; 2500; 1250; 625; 312; 156; 78 µg/mL) diluted in 10% DMSO (Dinâmica, Indaiatuba, Brazil) was combined with 100 µL of microbial suspension. Microbial suspensions were prepared from 24 h old colonies and standardized using the McFarland 0.5 scale (10^8^ CFU/mL), then diluted to 5 × 10^5^ CFU/mL in Mueller–Hinton broth (bacteria) or Sabouraud broth (fungi). The experiments were performed in triplicate.

Negative controls contained only microbial inoculum; sterility controls contained only culture medium; and positive controls included levofloxacin (0.25 mg/mL) for bacteria and terbinafine (0.40 mg/mL) for fungi. Plates were incubated at 37 °C for 24 h (bacteria) or 48 h (fungi). After incubation, 30 µL of 0.01% resazurin or 1% TTC (Sigma-Aldrich, Darmstadt, Germany) was added. Plates were incubated for an additional 2 h at 37 °C, and the MIC (minimum inhibitory concentration) was defined as the lowest extract concentration that inhibited microbial growth.

### 4.5. Thin-Layer Chromatography (TLC)

Fungal extracts were analyzed by TLC to identify major metabolite classes. Aliquots of 5 µL (5 mg/mL in methanol) were applied to silica gel plates (TLC aluminum sheets, Macherey-Nagel, 20 × 20 cm, silica gel 60, fluorescent indicator). The mobile phase consisted of dichloromethane–methanol–formic acid (90:8:2). Plates were developed, dried at room temperature, and examined under UV light at 254 nm. Chromophore compounds appeared as dark violet spots against a bright green background. Chemical developers were used to classify metabolites: aluminum chloride, ferric chloride, Dragendorff reagent, vanillin/H_2_SO_4_, and cerium sulfate.

### 4.6. Total Phenolic Content

Total phenolic content was estimated using the Folin–Ciocalteu method [60]. In test tubes, 40 µL of extract, 320 µL distilled water, and 40 µL Folin–Ciocalteu reagent were mixed and incubated for 6 min in the dark. Then, 640 µL of 10% Na_2_CO_3_ was added, mixed, and incubated for 1 h. Absorbance was read at 760 nm (UV-1280, Shimadzu, Kyoto, Japan). The results were expressed as mg of gallic acid equivalent per gram of extract (mg GAE/g), calculated from the linear regression standard curve y = 0.004x + 0.022 (R^2^ = 0.9991). Experiments were performed in triplicate, and statistical significance was assessed by an ANOVA followed by Tukey’s test (*p* < 0.05), using Minitab 18 software.

### 4.7. Fourier Transform Infrared Spectroscopy (FTIR)

The FTIR spectra of fungal extracts were acquired from 400 to 4000 cm^−1^ with 1 cm^−1^ resolution, averaging 70 scans, using an IRAffinity-1S spectrophotometer (Shimadzu, Kyoto, Japan) with attenuated total reflectance (ATR).

### 4.8. Ultra-High-Performance Liquid Chromatography (uHPLC)

The chromatographic profiles of the fungal extracts were obtained using uHPLC-DAD (Shimadzu Nexera XR—SPD-M20A) equipped with a Spherisorb ODS2 column (80 Å, 5 µm, 4.6 mm × 150 mm, Waters, Milford, CT, USA). The mobile phase consisted of acetonitrile–0.1% formic acid (80:20 *v*/*v*) at a flow rate of 0.7 mL/min in isocratic mode. Each run lasted 40 min, with the column temperature maintained at 40 °C. The samples (1 mg/mL in methanol) were filtered through PVDF membranes (25 mm diameter, 0.45 µm pore size), and 4 µL was injected.

### 4.9. UV–VIS Spectrophotometric Analysis

The UV-Vis absorption spectra of fungal extracts were recorded using a Shimadzu UV-1800 spectrophotometer, scanning from 200 to 800 nm with 1 nm resolution. Extracts were diluted to 30 µg/mL in methanol for analysis.

## 5. Conclusions

This study demonstrated that the application of the OSMAC strategy is an effective approach for exploring the metabolic potential of Amazonian filamentous fungi. By systematically varying the culture media, it was possible to modulate the production of secondary metabolites in *T. pinophilus* CCM-UEA-F0414 and *P. paxilli* CCM-UEA-F0591, resulting in significant differences in chemical profiles and antimicrobial activity. The fungal extracts displayed inhibitory effects against a wide range of clinically relevant pathogens, with the most notable activity observed against *C. albicans*, presenting MIC values of 78 µg/mL—a remarkable outcome for crude extracts.

Media such as YES and BDL enhanced both phenolic content and antimicrobial performance, underscoring the importance of culture optimization in microbial bioprospecting. Chemical analyses (TLC, FTIR, UV–Vis, and uHPLC-DAD) confirmed the presence of phenolic compounds, particularly caffeic and chlorogenic acids, as major constituents in the most active extracts. These compounds, traditionally associated with plant metabolism, were identified in fungal extracts, reinforcing the untapped biosynthetic potential of filamentous fungi.

Overall, our findings highlight Amazonian fungal biodiversity as a promising reservoir of bioactive molecules and validate the effectiveness of the OSMAC strategy in expanding chemical diversity. *T. pinophilus* CCM-UEA-F0414 and *P. paxilli* CCM-UEA-F0591 emerge as promising candidates for further purification, structural elucidation, and development of novel antimicrobial agents.

## Figures and Tables

**Table 1:** GenBank accession numbers for the fungal isolates used in this study.

Isolate	Species	Source	GenBank Accession Numbers		
			ITS	*tub*2	*rpb*2
CCM-UEA-F0414	*Talaromyces pinophilus*	*Myrcia guianensis*	PQ336018	-	PQ349268
CCM-UEA-F0591	*Penicillium paxilli*	*Aniba canelilla*	PQ336019	PQ349270	PQ349269

ITS: internal transcribed spacer; *tub2*: partial beta-tubulin gene; *rpb2*: second largest protein subunit of DNA-directed RNA polymerase II.

**Table 2:** Minimum inhibitory concentration (MIC) of ethyl acetate extracts from *T. pinophilus* CCM-UEA-F0414 and *P. paxilli* CCM-UEA-F0591 obtained after cultivation on BDL (B), modified ISP2 (I), YES (Y), modified Czapek (C), or malt (M) medium.

Microorganisms	Fungal Extracts (MIC, µg/mL)										Positive Controls	
	*T. pinophilus* CCM-UEA-F0414					*P. paxilli* CCM-UEA-F0591						
	B	I	Y	C	M	B	I	Y	C	M	Lev	Terb
*E. coli*	-	5000	-	-	5000	5000	-	-	5000	5000	0.061	NT
*B. subtilis*	-	1250	5000	5000	5000	5000	-	2500	-	2500	0.003	NT
*E. faecalis*	-	-	-	5000	-	5000	-	-	-	2500	1.953	NT
*K. pneumoniae*	-	-	-	5000	-	-	-	-	-	5000	250	NT
*P. aeruginosa*	5000	5000	5000	-	-	5000	2500	-	5000	5000	0.001	NT
*S. aureus*	-	5000	5000	156	-	2500	2.50	-	-	-	0.122	NT
*S.* *epidermidis*	-	5000	5000	1250	-	2500	313	-	-	5000	250	NT
*S. mutans*	-	-	-	2500	-	-	5000	5000	-	5000	0.0007	NT
*S. pyogenes*	1250	-	625	2500	1250	-	625	2500	-	-	0.003	NT
*S. enterica*	-	-	-	2500	-	5000	-	-	-	5000	0.0007	NT
*C. albicans*	156	156	78	313	156	78	-	5000	-	625	NT	50
*C. parapsilosis*	-	-	-	5000	-	-	-	2500	5000	5000	NT	400
*C. tropicalis*	2500	5000	5000	2500	5000	2500	625	1250	5000	2500	NT	25
*A. brasiliensis*	2500	-	2500	2500	-	-	-	-	-	-	NT	9.5 × 10^−7^

- = absence of antimicrobial activity. Lev = levofloxacin 0.25 mg/mL. Terb = terbinafine 0.40 mg/mL. NT = not tested.

**Table 3:** Quantification of caffeic acid and chlorogenic acid in fungal extracts produced by *T. pinophilus* CCM-UEA-F0414 and *P. paxilli* CCM-UEA-F0591 following cultivation in BDL (B), modified ISP2 (I), YES (Y), modified Czapek (C), or malt (M) medium, determined by uHPLC-DAD at 320 nm.

Isolate	Medium	Concentration (mg/g of Extract)	
		Caffeic Acid	Chlorogenic Acid
*T. pinophilus* CCM-UEA-F0414	B	1.7	0.43
	M	-	-
	I	-	-
	C	1.6	-
	Y	-	-
*P. paxilli* CCM-UEA-F0591	B	2.2	-
	M	-	-
	I	1.6	-
	C	0.6	-
	Y	0.6	-

- = not detected.

**Table 4:** Primers and PCR conditions used for molecular identification of fungi.

Locus ^1^	Primer	Sequence 5′–3′	T (°C) ^2^	Reference
ITS	*Its*1	TCCGTAGGTGAACCTGCGG	62	[51]
	*Its*4	TCCTCCGCTTATTGATATGC		
*tub*2	Bt-2a	GGT AAC CAA ATC GGT GCT GCT TTC	58	[52]
	Bt-2b	ACC CTC AGT GTA GTG ACC CTT GGC		
*rpb*2	RPB2-6F	TGG GGK WTG GTY TGY CCT GC	58	[53]
	fRPB2-7cR	CCC ATR GCT TGY TTR CCC AT		

^1^ ITS: internal transcribed spacer; *tub*2: partial beta-tubulin gene; *rpb*2: second largest protein subunit of DNA-directed RNA polymerase II. ^2^ T (°C): annealing temperature.

**Table 5:** Composition of liquid culture media used for fungal cultivation.

Culture Medium	Composition (g/L)
BDL (B)	Potato (200), dextrose (20), and yeast extract (2)
Malt (M)	Malt extract (20)
Modified ISP2 (I)	Corn Starch (4), yeast extract (4), and malt extract (10)
Modified CZAPEK (C)	D-glucose (20), Fe_2_(SO_4_)_3_ (10), K_2_HPO_4_ (1); MgSO_4_ (0.5), KCl (0.5), and yeast extract (2)
YES (Y)	Sucrose (150), yeast extract (20), and MgSO_4_ (0.5)

## Data Availability

Data are contained within this article and the Supplementary Materials.

## Supplementary Materials

The following supporting information can be downloaded at https://www.mdpi.com/article/10.3390/antibiotics14080756/s1, Table S1: GenBank accession numbers of sequences used in phylogenetic analyses of multiple genes. Figure S1: Comparative uHPLC-DAD chromatographic profile of chlorogenic acid standard (1 µg/mL, black line) and extract of *T. pinophilus* CCM-UEA-F0414 (red line). Figure S2: Comparison of uHPLC-DAD chromatographic profiles of caffeic acid standard (3 µg/mL, black line) and fungal extracts from *T. pinophilus* CCM-UEA-F0414 (red line—modified Czapek) and *P. paxilli* CCM-UEA-F0591 cultivated on different media (blue line—BDL; green line—modified ISP2; magenta line—modified Czapek; and cyan line—YES).
