# Supplementary material for: An OSMAC Strategy for the Production of Antimicrobial Compounds by the Amazonian Fungi Talaromyces pinophilus CCM-UEA-F0414 and Penicillium paxilli CCM-UEA-F0591
Source: Antibiotics (Basel). 2025 Jul 27;14(8):756. doi: 10.3390/antibiotics14080756 (PMC12383222; doi:10.3390/antibiotics14080756)
Supplement: Supplementary file 1 [file antibiotics-14-00756-s001.zip › antibiotics-3757637-supplementary.pdf]

Supplementary Material

Table S1. GenBank accession numbers of sequences used in the phylogenetic analyses of multiple genes.

Species Name	Specimen No.	GenBank accession no.[1]		
		ITS	***tub2***	***rpb2***
Penicillium citrinum	CBS 139.45^T	MH856132	GU944545	JF417416
Penicillium copticola	CBS 127355^T	JN617685.	JN606817	JN606599.
Penicillium dokdoense	CNUFC DDS11-1^T	MG906868	MH243037	
Penicillium gorlenkoanum	CBS 408.69^T	GU944581	GU944520	JN606601
Penicillium hetheringtonii	CBS 122392^T	GU944558	GU944538	JN606606
Penicillium paxilli	CBS 360.48^T	GU944577	-	JN606610
Penicillium shearii	CBS 290.48^T	GU944606	-	JN121482
Penicillium sizovae	CBS 413.69^T	GU944588	GU944535	JN606603
Penicillium steckii	CBS 260.55^T	GU944597	GU944522	JN606602
Penicillium sumatraense	CBS 281.36^T	GU944578	-	-
Penicillium terrigenum	CBS 127354^T	JN617684	JN606810	JN606600
Penicillium tropicoides	CBS 122410^T	GU944584	JN606810	JN606608
Penicillium tropicum	CBS 112584^T	GU944582	GU944532	JN606607
Talaromyces aculeatus	NRRL 2129^T	MH793036	-	MH793099
Talaromyces adpressus	CBS 140620^T	KU866657	-	KU867001
Talaromyces-angelicae	KACC 46611	-	-	KX961275
Talaromyces annesophieae	CBS 142939^T	NR_170732	-	MN969199
Talaromyces apiculatus	CBS 312.59^T	KF741983	-	KM023287
Talaromyces beijingensis	CBS 140617^T	KU866649	-	KU866993
Talaromyces brevis	CBS 141833^T	MN864269	-	MN863328
Talaromyces dimorphus	NN072337	KY007095	-	KY112593
Talaromyces domesticus	NRRL 58121^T	MH793055	-	MH793118
Talaromyces fuscoviridis	CBS 193.69^T	NR_153227	-	MN969156
Talaromyces lentulus	NN071323	KY007088	-	KY112586
Talaromyces liani	CBS 225.66^T	JN899395	-	KX961277
Talaromyces mae	NN071327	KY007089	-	KY112587
Talaromyces malicola	NRRL 3724^T	MH909513	-	MH909567
Talaromyces pinophilus	CBS 631.66^T	JN899382	-	KM023291
Talaromyces pratensis	NRRL 62170^T	MH793075	-	MH793139
Talaromyces sayulitensis	DTO 245-H1^T	OK339606	-	MN969146
Talaromyces soli	NRRL 62165^T	MH793074	-	MH793138
Talaromyces tumuli	NRRL 62151^T	MH793071	-	MH793135
Talaromyces versatilis	AS3.3708	MK837960	-	MK837968

[1]GenBank - Internal transcribed spacer regions (ITS), β-tubulin (*tub2*), and the second largest protein subunit of DNA-directed RNA polymerase II (*rpb2*).
-: Sequence not available.

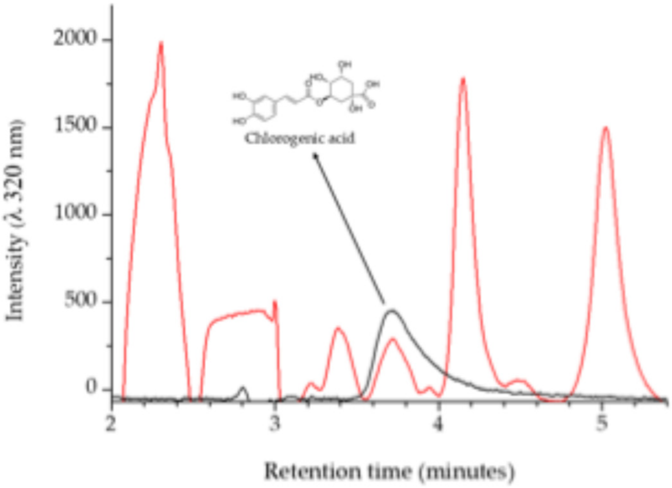

Figure S1. Comparative uHPLC-DAD chromatographic profile of chlorogenic acid standard (1 µg/mL, black line) and the extract of *T. pinophilus* CCM-UEA-F0414 (red line).

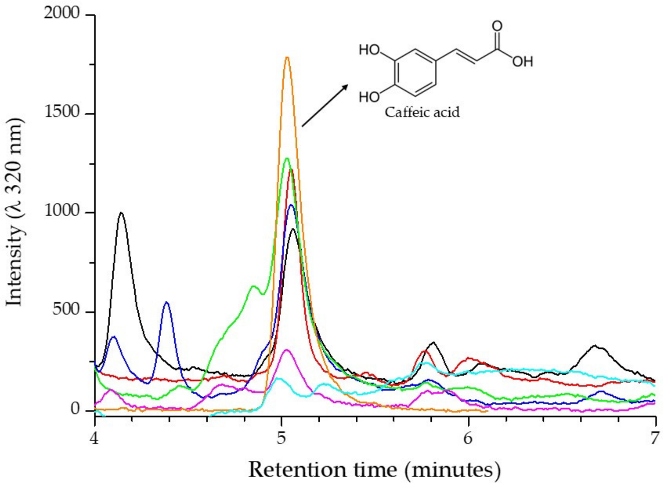

Figure S2. Comparison of uHPLC-DAD chromatographic profiles of caffeic acid standard (3 µg/mL, black line) and fungal extracts from *T. pinophilus* CCM-UEA-F0414 (red line - modified Czapek) and *P. paxilli* CCM-UEA-F0591 cultivated on different media (blue line – BDL; green line - modified ISP2; magenta line - modified Czapek; and cyan line - YES).
